# Alprazolam Detection Using an Electrochemical Nanobiosensor Based on AuNUs/Fe-Ni@rGO Nanocomposite

**DOI:** 10.3390/bios12110945

**Published:** 2022-10-31

**Authors:** Emadoddin Amin Sadrabadi, Fatemeh Khosravi, Ali Benvidi, Amin Shiralizadeh Dezfuli, Pouria Khashayar, Patricia Khashayar, Mostafa Azimzadeh

**Affiliations:** 1 Department of Chemistry, Faculty of Science, Yazd University, Yazd 8915818411, Iran; 2 Department of Medical Biotechnology, School of Medicine, Shahid Sadoughi University of Medical Sciences, Yazd 8915173143, Iran; 3 Center of Excellence in Electrochemistry, Faculty of Chemistry, University of Tehran, Tehran 1439957131, Iran; 4 Ronash Technology Pars Company, Tehran 1439817435, Iran; 5 Institute of Cardiovascular and Medical Sciences, University of Glasgow, Glasgow G3 8QP, UK; 6 Center for Microsystem Technology, Imec and Ghent University, 9000 Gent, Belgium; 7 Medical Nanotechnology & Tissue Engineering Research Center, Yazd Reproductive Sciences Institute, Shahid Sadoughi University of Medical Sciences, Yazd 8916877391, Iran; 8 Stem Cell Biology Research Center, Yazd Reproductive Sciences Institute, Shahid Sadoughi University of Medical Sciences, Yazd 8916877391, Iran

**Keywords:** nanobiosensor, alprazolam, AuNUs, rGO, electrochemical pretreatment

## Abstract

Despite all the psychological advantages of alprazolam, its long list of toxic properties and interactions has caused concern and highlighted the need for a reliable sensing method. In this study, we developed a simple, highly sensitive electrochemical nanobiosensor to determine the desirable dose of alprazolam, averting the undesirable consequences of overdose. Gold nanourchins (AuNUs) and iron-nickel reduced graphene oxide (Fe-Ni@rGO) were immobilized on a glassy carbon electrode, which was treated beforehand. The electrode surface was characterized using cyclic voltammetry, Fourier transform infrared spectroscopy, scanning electron microscopy/energy-dispersive X-ray spectroscopy, and differential pulse voltammetry. The fabricated sensor showed two linear ranges (4 to 500 µg L^−1^ and 1 to 50 mg L^−1^), low limit of detection (1 µg L^−1^), high sensitivity, good repeatability, and good recovery. Increased –OH and carboxyl (-COOH) groups on the electrode surface, resulting in improved the adsorption of alprazolam and thus lower limit of detection. This nanobiosensor could detect alprazolam powder dissolved in diluted blood serum; we also studied other benzodiazepine drugs (clonazepam, oxazepam, and diazepam) with this nanobiosensor, and results were sensible, with a significant difference.

## 1. Introduction

Benzodiazepines (BZDs) are a class of psychoactive drugs known for their depressant effects on the central nervous system (CNS) [1]. They became popular in the 60s and 70s due to the influence of The Rolling Stones and numerous Hollywood movies sensationalizing Valium (diazepam). Individuals with anxiety and difficulty sleeping were encouraged to take BZDs, which contributed to the popularity of the medication [2]. Alprazolam (or Xanax) is the most commonly prescribed BZD, generally for short-term treatment of psychiatric disorders [3]. “Misuse liability” due to continued use of the drug for longer periods, however, can be addictive [4].

The coronavirus pandemic had a negative impact on mental health, leading to a significant increase in the number of patients and even healthcare professionals taking BZDs [5]. BZDs such as Alprazolam are not only addictive in long-term usage; they may also result in severe toxicity when used in combination with alcohol/opiates. Alprazolam is also reported to be significantly more toxic than other BZDs. The most common side effects of alprazolam include depression, sedation, fatigue, ataxia, amnesia, dysarthria, headache, slurred speech, poor concentration, hypersensitivity, irritability, and memory impairment [4]. Due to these pernicious effects, developing a simple and highly sensitive method to assess a desirable dose of alprazolam can help prevent possible damages to the body.

Existing detection technologies for BZDs include high-performance liquid chromatography (HPLC) [6], gas chromatography (GC) [7], ultraviolet-visible (UV-vis) spectroscopy [8], photometrics (nuclear magnetic resonance and UV-vis), and electrochemical sensors. Gas chromatography connected to mass spectrometry (GC-MS) was considered for the reference method because of its high sensitivity and specificity. It was also concluded to be the best solution for challenging cases [9]. However, the necessity of sample preparation complicated the automation possibilities of this technique [10,11]. Due to the thermolability of most BZDs, resulting in their rapid degradation in the absence of prior derivatization, this method is not appropriate for emergency toxicological analysis. HPLC, on the other hand, results in no derivatization reactions and thus no thermal degradation of the molecules, and needs less complicated sample preparation [12,13]. In this technique, the analysis was performed with UV spectrophotometry using a photodiode-array detector with a short turnaround time, which turned this method into a suitable technique for determining BZDs in biological samples and emergency situations [14]. Despite all these advantages, HPLC still needs sophisticated equipment and has low selectivity in comparison to GC [9,15]. As a result, HPLC coupled with mass spectrometry (HPLC-MS) was the method of choice for the identification and quantification of BZDs in very low circulating concentrations [12,16,17]. Despite the thin-layer chromatography (TLC) method being quite fast (about half an hour), its results are not reliable due to the lack of specificity and sensitivity. This method is thus less common in emergency toxicology cases [18,19]. Some claim UV-vis spectroscopy can detect BZDs with a lower limit of detection and subsequently overcome the limitations of most immunoassays [20]. However, compared to other methods for BZD characterization, this technique is only used in certain laboratories [9].

Electrochemical sensors are becoming more popular due to their simplicity, high sensitivity, excellent selectivity, low cost, fast response, and ease of operation and sample-preparation methods [21,22]. Glassy carbon electrodes (GCEs) are the most frequently used electrodes for those biosensors due to their wide potential window, low background noise, easy surface modification techniques, and low cost [23,24,25,26].

The pretreatment process of the carbon electrodes helped improve their sensitivity and selectivity by increasing the charge that carries functional groups on their surface. These functional groups, which contain oxygen, not only increased the number of active sites in the redox system but also helped with the proton or electron exchange between the surface and the redox system. As a result, the electrodes showed more sensitivity towards a specific analyte. In addition to being cost-effective and simple, electrochemical pretreatment (compared to methods such as mechanical polishing; solvent cleaning; and vacuum heat, laser-based thermal, microwave plasma, and radio-frequency plasma treatment) is reported to have the most significant effect on surface microstructure and features [27].

Several studies have been aimed at detecting alprazolam using electrochemical techniques [21,28,29,30]; the majority of them, however, have failed to achieve the required limits of detection. Some have shown that use of nanomaterials can help accomplish a lower limit of detection and a wider linear range [31] through increase of surface-area-to-volume ratio, conductivity, and interaction with the target analyte [32].

**Figure 1:**
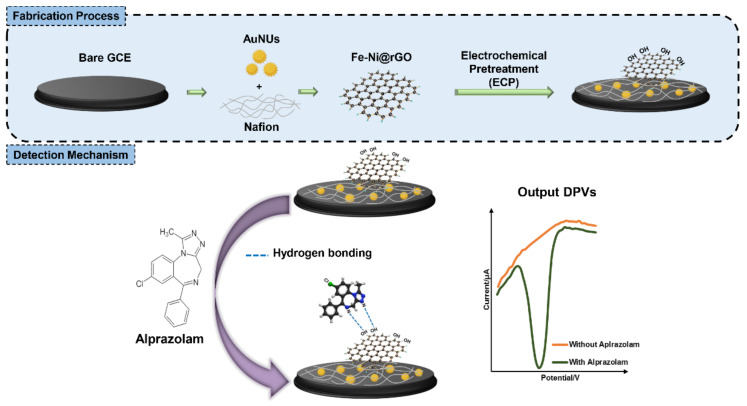
Schematic of the fabrication and measurement of the electrochemical nanobiosensor for alprazolam (Xanax) assessment.

In this study, therefore, we proposed the fabrication of an electrochemical nanosensor as a screening tool for the determination of alprazolam levels using a combination of nanomaterials, namely gold nanourchins (AuNUs) and Fe-Ni modified graphene oxide (Fe-Ni@rGO). Nafion, an electrically conductive perfluorinated membrane containing a hydrophobic matrix, hydrophilic channels and pores [33], was used to stabilize gold nanourchins on the electrode surface. Figure 1 (top section) is a schematic overview of the fabrication process using the nanomaterials along with pretreatment of a working electrode. The bottom section illustrates the working mechanism of the electrochemical nanobiosensor for alprazolam.

## 2. Materials and Methods

### 2.1. Chemicals

Nanomaterials, including iron- and nickel-modified reduced graphene oxide (Fe-Ni@rGO), gold nanourchins (AuNUs), silver-conjugated graphene quantum dots (GQD-Ag), and cadmium sulfide quantum dots (CdSQDs), were purchased from Ronash Technology Pars Co., Tehran, Iran (www.aminbic.com, accessed on 20 February 2022). Fe-Ni@rGO layers (5 µ m) were composed of a composite produced by anchoring nickel ferrite (NiFe_2_O_4_) on a graphene oxide surface. AgQDs (10 nm) were placed on the surface of graphene oxide (30–40 nm) with 1-2 layers. CdSQDs (3–6 nm) are semiconductor compounds that are dispersed in water. AuNUs (about 60 nm) are also water-dispersed compounds. Alprazolam powder was ordered from Tehran Darou Co., Tehran, Iran (www.tehrandarou.com, accessed on 20 February 2022). Potassium ferricyanide (K_3_[Fe(CN)_6_]), potassium ferrocyanide (K_4_[Fe(CN)_6_]), potassium chloride (KCL), sodium dihydrogen phosphate (NaH_2_PO_4_), disodium hydrogen phosphate (Na_2_HPO_4_), boric acid (H_3_BO_3_), acetic acid (CH_3_COOH), sodium hydroxide (NaOH), Nafion (C_7_HF_13_O_5_S·C_2_F_4_), ethanol (C_2_H_5_OH), and aluminum oxide powder (Alumina) were purchased from Sigma, St. Louis, MO, USA. All solutions were prepared with double-distilled water in a clean environment and either autoclaved or filtered for sterilization based on their heat tolerance. Solutions were prepared fresh daily just before usage and used only one time each.

In order to make a Britton–Robinson buffer (BRB), a mixture of 0.04 M boric acid, 0.04 M phosphoric acid, and 0.04 M acetic acid was adjusted to pH 9.0 with 0.2 M sodium hydroxide using a Metrohm model 691 pH/mV meter. For preparation of a phosphate buffer solution (PBS), 3.394 g sodium dihydrogen phosphate (NaH_2_PO_4_) and 20.214 g disodium hydrogen phosphate (Na_2_HPO_4_) were mixed in 800 mL miliQ water, then adjusted to pH 7.4 using NaOH and distilled water until the volume of 1000 mL was achieved. Human serum samples were obtained from a volunteer who was informed about the purpose of the study and how the sample would be processed for the nanobiosensor measurement experiments.

### 2.2. Electrode Surface Cleaning and Modification

The cleaning process commenced with polishing of a working electrode in an 8-like shape using a cloth soaked in 1.0 μm and 0.05 μm alumina–water slurry to remove any surface contamination. Next, the GCE was washed several times with ddH_2_O and then sonicated for 5 min in a 1:1 water and ethanol solution to remove possible residuals. As the final step, it was again washed with ddH_2_O to assure its cleanliness.

To improve performance, the working electrode was also electrochemically pretreated (ECP) to increase the number of functional groups needed for better attachment of nanomaterials. In this regard, the electrode was anodized for 600 s at a potential of +1.6 V, followed by an optimal cathodization for 10 seconds at a potential of −1.2 in PBS solution (pH 7.4). The successful achievement of a clean surface was confirmed by a CV test in a solution of 5.0 mM K_3_[Fe (CN)_6_]/K_4_[Fe (CN)_6_] containing 0.1 M KCl.

In the next step, nanomaterials were deposited onto the working electrode. The used nanomaterials were first sonicated for five minutes to achieve a dispersed solution with a lower rate of agglomeration. In one attempt, a 4 µL drop of AuNU solution (30.0 µg·mL^−1^ in 0.5 wt% Nafion solution) was deposited on the bare, cleaned GCE; after the GCE became semi-dried, 6 µL Fe-Ni@rGO solution (30.0 mg·mL^−1^ in H_2_O) was deposited on the electrode and left until completely dried. Subsequently, the nano-modified electrode was flushed with ddH_2_O to remove any unattached nanomaterials from the surface. Depositions were performed while the electrode was placed in a clean container to prevent possible contamination and allow the electrode to dry under stable conditions at room temperature.

In order to test the effect of quantum dots, a combination of two quantum dots, 3 µL of GQD-Ag (4 mg·mL^−1^ in H_2_O), and 3 µL of CdSQD (3 mg·mL^−1^ in H_2_O) was dropped on the surface of the electrode, and again, the electrode was left until completely dried. It was then washed twice using ddH_2_O. Afterwards, a drop of 30.0 mg·mL^−1^ Fe-Ni@rGO solution was dropped on the surface of the QD-modified electrode and kept until the electrode had completely dried. This was followed by washing with ddH2O to remove possible unattached nanomaterials.

### 2.3. Optimization Method

Nanomaterial selection was optimized through a series of experiments to improve performance of the alprazolam biosensor. The arrangement of the nanomaterial was first optimized through Design of Experiment (DOE) studies. Full Factorial Design (FFD) in Minitab^®^ Statistical Software (Minitab LLC, State College, PA, USA) (version 21.1) was used to determine effective factors and increase current density in bare and nano-modified working electrodes. The performance of Fe-Ni@rGO, AuNUs, GQD-Ag, and CdSQDs were compared in that regard. The effect of each nanomaterial was also assessed using cyclic voltammetry (CV) after every step to assure quality of the process as well as functionality of the biosensor. CVs in general were used to optimize concentration and volume of deposited nanomaterials, whereas the peak current of recorded CV curves was used to compare efficacy of nanomaterials.

### 2.4. Electrochemical Measurements

Electrochemical measurements were conducted at standard laboratory temperature (25 ± 1 °C) and humidity using a potentiostat/galvanostat model PGSTAT 302 N from Eco Chemic Co., (Utrecht, The Netherlands) connected to a computer with NOVA software ver. 1.7. A three-electrode system consisting of a GCE (with a diameter of 1.5 mm) as a working electrode, Ag/AgCl as a reference, and platinum as a counter electrode was used. CV was performed in a solution of 5.0 mM K_3_[Fe (CN)_6_]/K_4_[Fe (CN)_6_] containing 0.1 M KCl at a potential range of −0.17 to 0.6 V and a scan rate of 0.02 V/s.

Alprazolam with different concentrations was used in order to plot calibration curves. Thereafter, the signal was measured following adsorption of alprazolam on the surface using differential pulse voltammetry (DPV). DPV readings were carried out in BRB solution with a pH level of 9.0, in the potential range of −0.9 V to −1.2 V, 50 mV step potential, 25 mV amplitude, and 0.05 s modulation time. All DPV measurements were subjected to baseline correction for better comparison.

### 2.5. Characterization and Assessment Methods

In addition to electrochemical measurements, other features of the nanomaterial and the biosensor were tested using supplementary techniques. Decoration and dispersion of nanomaterials on the GCE surface were evaluated with a Scanning Electron Microscope (SEM) using SIGMA instrument model VP FE-SEM (Zeiss SIGMA, Oberkochen, Germany). Energy Dispersive Spectroscopy (EDS) was used for elemental analysis of the surface using the SEM instrument. Fourier transform infrared (FTIR) spectroscopy was performed to assess the chemical bonds within and between applied nanomaterials using an Avatar 360 instrument (Thermal Nicolet, Nicolet, QC, Canada).

## 3. Results and Discussion

In recent years, more attention has shifted towards the use of conductive nanomaterials (CNMs) in biosensing studies due to their higher surface-to-volume ratio and greater electron-signal transfer rate. This is mainly because their increased conductivity and electrocatalytic properties help magnify electrochemical signals and thus sensitivity and selectivity of the biosensor [34,35].

The proposed biosensor in this article benefited from those CNMs through the application of a combination of nanomaterials, each successfully shown to improve the performance of biosensors in previous studies. In other words, those nanomaterials helped the biosensor achieve lower limits of detection by expanding surface area and conductivity of the working electrode.

Nanomaterials of the graphene family, such as graphene, graphene oxide, reduced graphene oxide, and modified/functionalized graphene oxide, are widely used in biosensors, mostly because of their high specific surface area and vast chemical modification potentials. In this platform, modified/functionalized graphene oxide (Fe-Ni@rGO) increased the specific surface area on the electrode, providing an infrastructure for vast chemical modification potentials [36,37,38,39,40,41,42]. Coupling reduced graphene oxide with gold nanoparticles, another common nanomaterial in fabrication of electrochemical nanobiosensors, added advantages such as increased conductivity, compatibility, and surface-area-to-volume ratio [43,44]. Gold nanomaterials display good biocompatibility, benign biocatalytic properties, and excellent biosensing properties, which make them a good candidate for biological detection systems such as biosensors [45]. Quantum dots, another group recently becoming more and more popular in biosensing studies, were also added because of their large surface area, excellent biocompatibility, quantum confinement, edge effects, and abundant sites for chemical modification [46,47].

### 3.1. Optimization Results

**Figure 2:**
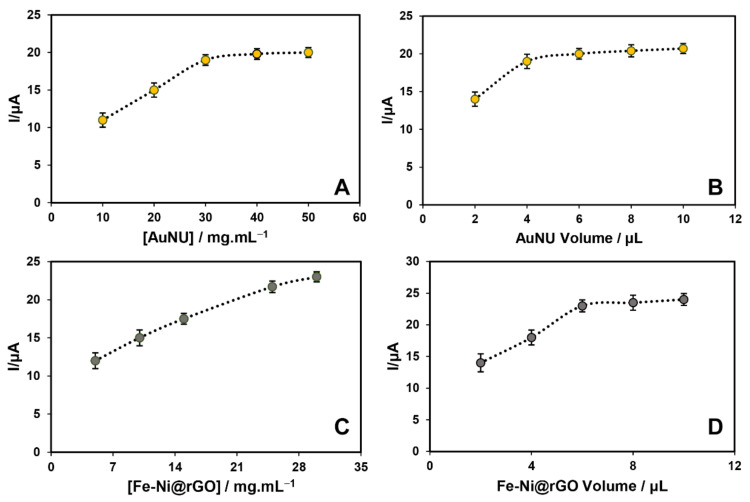
Optimization of concentration (**A**) and volume (**B**) of deposited suspension of AuNUs as well as concentration (**C**) and volume (**D**) of Fe-Ni@rGO. (n = 3).

A literature review performed by our group at the beginning of this study reported Fe-Ni@rGO, AuNUs, GQD-Ag, and CdSQD as the nanomaterials of choice to enhance the sensitivity of the biosensor. In the next step, possible combinations of those materials were compared in order to achieve the best outcome or biosensing performance. In that regard, calibration models could have been obtained either through one-factor-at-a-time (OFAT) or a holistic approach based on DoE. We selected the latter, as DOE is an efficient and systematic way to examine the relationship between multiple input and output variables in a structured manner [48,49]. To the best of our knowledge, this is the first time that this type of systematic analysis has been carried out to determine an optimized value for a nanobiosensor for alprazolam. Based on the DOE results (shown in Supplementary Materials), we decided to continue our experiment with Fe-Ni@rGO and AuNUs. The optimization process of these nanomaterials with differential pulse voltammetry (DPV) after three repetitions is shown in Figure 2.

The best combination in these tests was determined to be the one resulting in the highest increase in final DPV signal currents. Using these criteria, a combination of AuNUs and Fe-Ni@rGO was suggested as the best CNM to enhance the sensitivity of the biosensor. As a result, which can be seen in Figure 1, this combination was used in the fabrication of the final biosensor.

In the next step, certain experimental conditions, including concentration of nanomaterials and their volume at the electrode surface, were optimized. In that regard, various concentrations and volumes of AuNUs were applied to the clean GCE surface. CV analysis revealed a sharp increase in the response of modified electrodes with any increase in AuNU concentration from 0.01 to 0.03 mg·mL^−1^, followed by a plateau phase between 0.03 and 0.05 mg·mL^−1^ (Figure 2A). As for the volume of gold nanoparticles on the electrode surface, similarly, a significant increase in the final signal was noted between the volumes of 2 and 4 μL, followed by no change regardless of any increase in the volume between 4 and 10 μL (Figure 2B). The highest currents were therefore obtained using 4 µL of 0.03 mg·mL^−1^ AuNUs.

The same process was used to measure the optimal concentration and volume of Fe-Ni@rGO. As can be seen in Figure 2, with any increase in the Fe-Ni@rGO concentration, an increase in current occurred as expected; this trend had a steeper slope in the range of 6 to 25 mg·mL^−1^ and showed a more stable trend in the range of 25 to 30 mg·mL^−1^. The volume of Fe-Ni@rGO nanoparticles on the electrode surface was then determined. Figure 2D shows the increasing trend of the signal from 2 to 6 µL and the stabilization of the process from 6 to 10 µL. As can be seen in Figure 2C,D, 6 µL of a 30 mg·mL^−1^ Fe-Ni@rGO suspension resulted in the best outcome and therefore was used in the following attempts to prepare the nanobiosensor. It should be noted that in this section, due to high variety of nanomaterials, an attempt was made to use small amounts of volume in order to modify the surface to prevent agglomeration and reduce interference effects. It is also worth mentioning that higher volumes were investigated and the values introduced as optimal values were lowest volume and concentration.

### 3.2. Characterization Results

**Figure 3:**
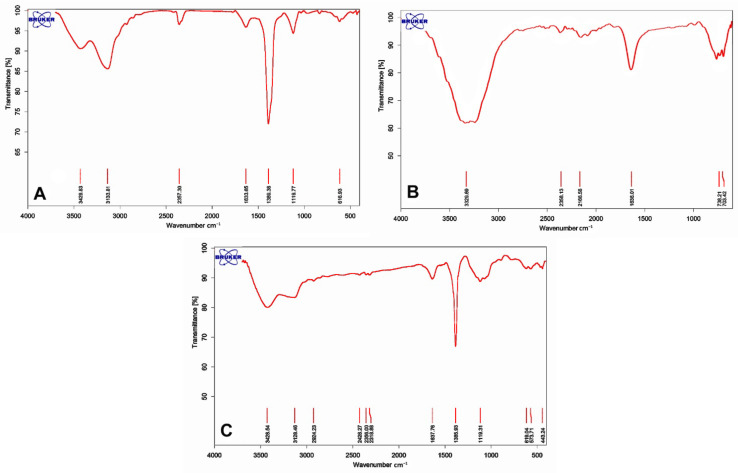
FTIR of (**A**) Fe-Ni@rGO, (**B**) AuNUs, and (**C**) AuNUs/Fe-Ni@rGO.

The FTIR spectra of Fe-Ni@rGO in Figure 3A display five characteristic peaks at 616, 1633, 1389, 3133, and 3429 cm^−1^, corresponding to Fe-O vibration in α-Fe_2_O_3_, stretching vibration of C=C, stretching vibration of epoxy (C-O) groups, sp^2^ C-H bond stretch, and stretching vibration of water molecules, respectively. The FTIR diagram of AuNUs (Figure 3B) shows strong bands at 602, 1096, 1201, 1388, and 1636 cm^−1^. The C-S stretching vibration was located at 750–550 cm^−1^. Bands at 1636 cm^−1^ were attributed to the vibrations of C=C double bonds in the AuNUs. The broad absorption band in the region of 3510–3235 cm^−1^ was attributed to the stretching vibration of –OH, H_2_O, and –NH. Figure 3C shows a combination of FTIR spectra, confirming the presence of Fe-Ni@rGO and AuNUs.

**Figure 4:**
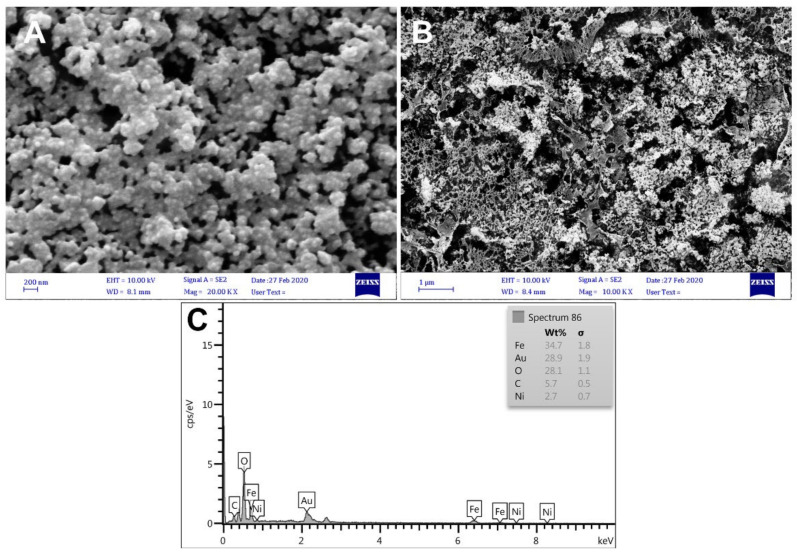
(**A**,**B**) SEM and (**C**) EDS of the GCE after modification with AuNUs/Fe-Ni@rGO.

In this experiment, morphology of modified electrodes was studied using SEM. Figure 4A shows the morphology and spectrum of Au nanourchins. It is evident that these urchin-like structures exhibit a broader spectrum than spherical NPs, mainly due to their shape and size. In Figure 4B, the external-texture SEM image of the Fe-Ni@rGO nanocomposite is illustrated. The presence of a sheet-like, curved, and wrinkled graphene oxide film and Fe nanoparticles in the form of spherical particles dispersed on the sheets was evident. These images confirmed high porosity of the developed surface, which helped increase the surface-to-volume ratio, and thus the biosensor’s sensitivity, significantly.

EDS analysis was then used to confirm the presence of nanomaterials used in surface modification. EDS analysis of GCE/AuNUs/Fe-Ni@rGO (Figure 4C) highlighted the existence of elements such as iron, nickel, carbon, and oxygen atoms associated with Fe-Ni@rGO and gold atoms associated with AuNUs on the surface.

### 3.3. Electrochemical Behavior

**Figure 5:**
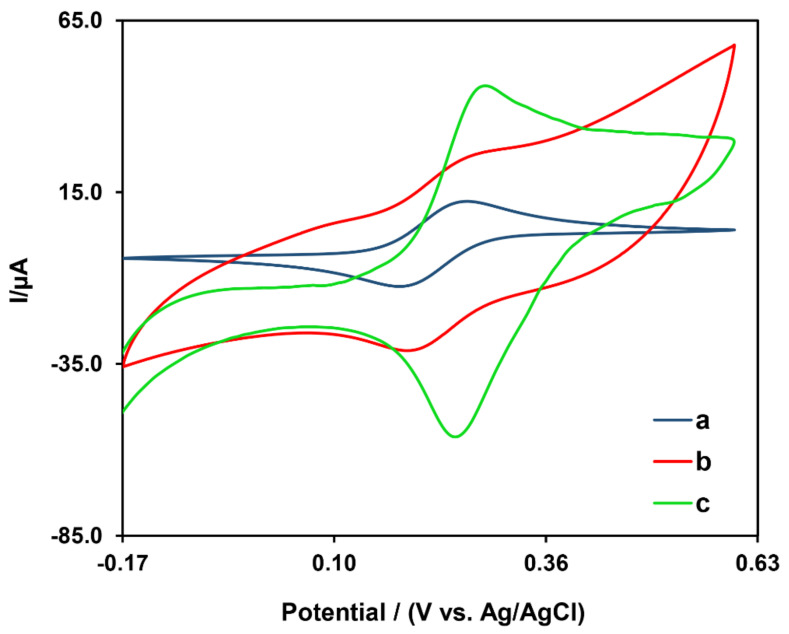
Cyclic voltammetry of bare GCE (a), GCE coated with Fe-Ni@rGO (b), and GCE modified with Fe-Ni@rGO/AuNUs (c) in a solution of 5.0 mM [Fe (CN)_6_]^3−/4−^ containing 0.1 M KCl.

Electrochemical characteristics of bare GCE and nano-modified electrodes were assessed using CV and DPV techniques. Figure 5 shows CV curves of the working electrode (GCE) in 0.5 mM [Fe(CN)_6_]^3−/4−^ solution for different steps of the fabrication of the designed electrochemical nanobiosensor (Bare GCE, Fe-Ni@rGO/GCE, and Fe-Ni@rGO/AuNP/GCE).

As can be seen in Figure 5′s CV curves, a significant increase in voltammogram peak current after coating of the bare electrode surface (a) with Fe-Ni@rGO (b) was noted. After the addition of a combination of AuNUs/Fe-Ni@rGO (c), on the other hand, a higher peak current, along with a proper redox peak, was reported. From these results, it can be concluded that the use of those nanomaterials helped increase conductivity and perhaps surface area of the electrode. Those nanomaterials, therefore, can help enhance sensitivity of the final alprazolam nanobiosensor.

**Figure 6:**
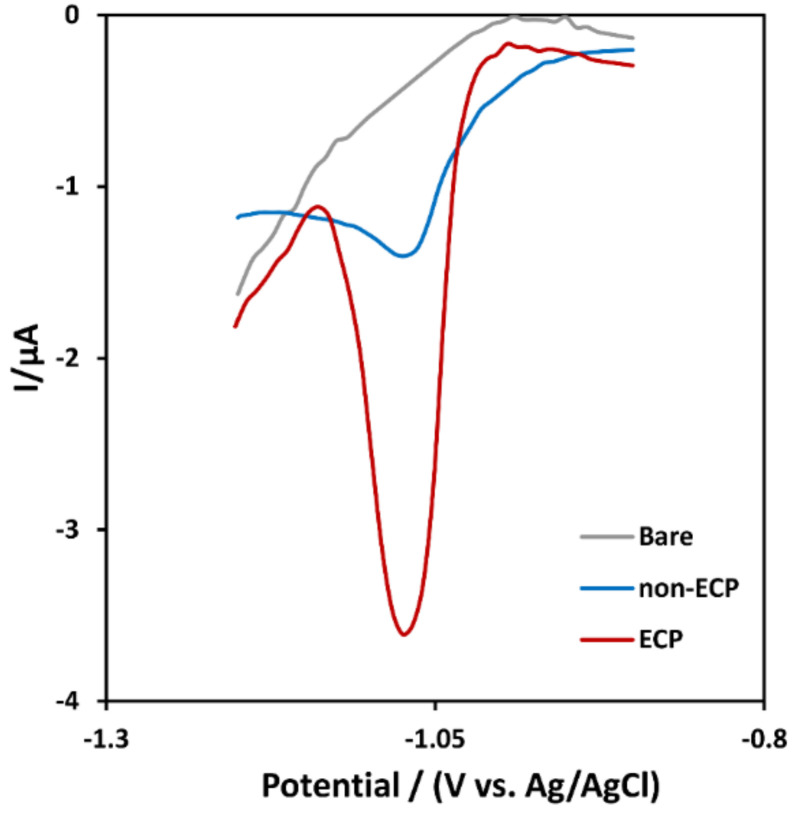
DPV of Bare, non-ECP, and ECP of a modified electrode for 500 μg/L of alprazolam in Britton–Robinson buffer solution (BRB) with a pH of 9.0.

Due to its high attraction to forming hydrogen bonds, alprazolam formed strong bonds with functional groups in Fe-Ni@rGO after electrochemical pretreatment (ECP), resulting in a lower detection limit [21,48,50,51]. As shown in Figure 6, the bare electrode diagram did not show a specific peak while measuring certain amounts of alprazolam (500 µg·mL^−1^); the peak current of ECP nanobiosensor, on the other hand, was significantly higher than that of the non-ECP nanobiosensor. Increasing the number of carboxyl and hydroxyl groups (-COOH and -OH), therefore, improved the capability of the nanobiosensor to detect alprazolam as more drug was adsorbed on the surface through hydrogen bonds.

**Figure 7:**
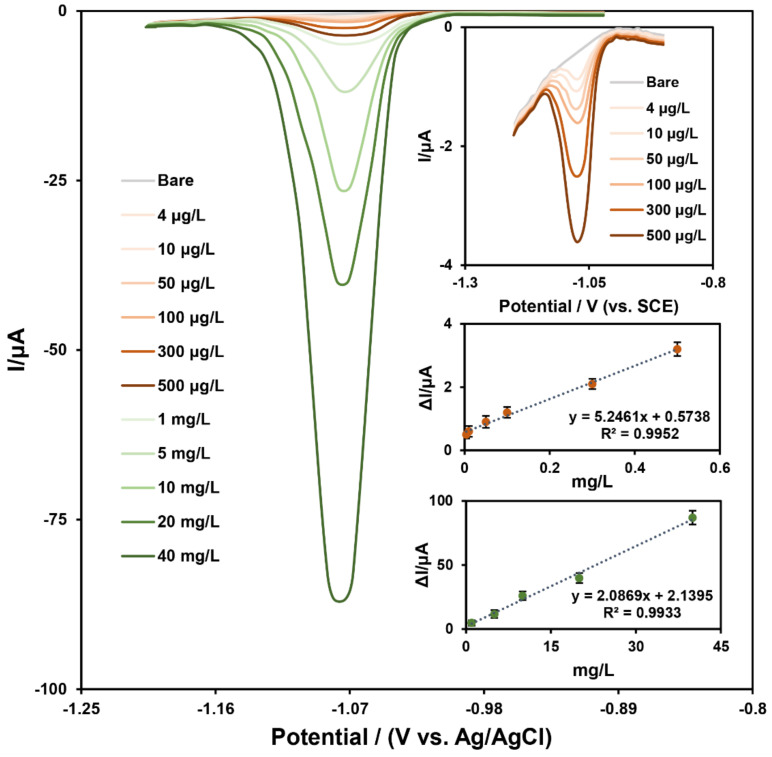
DPV of different concentrations of alprazolam in Britton–Robinson buffer solution (BRB) with a pH of 9.0 (two bottom insets show the calibration curves). (n = 3).

As a final readout signal, DPV was used to measure various concentrations of alprazolam under optimal conditions and determine analytical performance of the prepared nanobiosensor. Figure 7 illustrates the recorded DPV curves after addition of different concentrations of alprazolam to the GCE/AuNUs/Fe-Ni@rGO electrode in the Britton–Robinson buffer solution (pH = 9.0).

Two linear ranges were produced based on the calibration plot of cathodic peak current versus alprazolam concentrations: the first from 4 to 500 μg L^−1^ and another from 1 to 40 mg L^−1^. As expected, any increase in the concentration of alprazolam was associated with a higher surface adsorption rate and thus larger signals [21]. The nanobiosensor also exhibited a low limit of detection of 1 µg L^−1^ for detecting alprazolam.

Sensitivity of the biosensor was shown to vary based on the method used to adsorb alprazolam on the surface of the modified electrode. Higher sensitivity over a linear range was noted, with alprazolam being adsorbed as a monolayer on the modified electrode surface (Figure 7). When it was adsorbed as a multilayer at higher concentrations, however, lower sensitivity was noted over the linear range [48,50,51].

Table 1 compares certain analytical features of the current nanobiosensor with that of previous nanobiosensors. Among them, the proposed alprazolam sensor shows a wider linear range and the lowest detection limit. Similar to our sensor, Ashrafi et al. used a unique conductive nano-ink based on silver nanoparticle-nitrogen doped graphene quantum dots (Ag/N-GQD) to measure BZDs (such as alprazolam, chlordiazepoxide bis, diazepam, oxazepam, and clonazepam). This biosensor could detect alprazolam with a linear range of 56–156 μM (DPV) and a lower limit of detection of 56 μM (DPV) [52]. The better analytical performance of our biosensor can be attributed to the electrochemical pre-preparation technique applied in this project. This procedure increased the number of carboxyl functional groups on the electrode surface, which resulted in improved absorption of alprazolam molecules and therefore better performance. Another study by Boonmee and colleagues was conducted on the same type of electrode (GCE) but without using any nanomaterials. In that study, the GCE, which was pretreated electrochemically, demonstrated good adsorption and electrochemical reduction of alprazolam, confirmed with SEM/energy dispersive X-ray spectroscopy, Fourier transform infrared spectroscopy, CV, and electrochemical impedance spectroscopy. The fabricated sensor showed a quantification limit of 0.1 mg L^−1^, a detection limit of 0.03 mg L^−1^, and two linear ranges: 0.1 to 4 and 4 to 20 mg L^−1^ [21].

Hall et al. used HPLC for the detection of alprazolam in blood samples. They reported a limit of detection of 18 ng/mL and a retention time of 6.6 min for alprazolam [53]. In comparison to our biosensor, their detection method had a higher limit of detection (lower sensitivity) and needed more time to finalize the detection process. On the other hand, a large, expensive HPLC instrument and columns were also required, which reduced the favorability of the method for future medical applications.

**Table 1:** Comparison of nanobiosensor features and analytical performance to others published about alprazolam quantification.

Electrode	Detection Method	Linear Range	LOD	Ref.
Electrochemical Methods				
GCE	EIS	0.1 to 4 and 4 to 20 mg/L	0.03 mg/L	[21]
Carbon paste electrode	DPV			[28]
Boron-doped diamond electrodes	DPV	8 × 10^−7^–1 × 10^−4^ M	6.4 × 10^−7^ M	[29]
PVC membrane and carbon paste electrodes	Potentiometric	PVC: 1.0 × 10^−6^–1.0 × 10^−2^ M CPE: 1.0 × 10^−6^–1.0 × 10^−2^ M	1.0 × 10^−6^ M	[49]
Meniscus-modified silver solid amalgam electrode	DPV	0.185–30.9 mg/L	0.155 mg/L	[54]
Gold electrode	DPV SWV	DPV: 56–156 μM SWV: 73–192 μM	LLOQ of DPV: 56 μM LLOQ of SWV: 73 μM	[52]
GCE	DPV	4 to 500 µg L^−1^ and 1 to 50 mg L^−1^	1 µg L^−1^	This work
**Other methods**				
-	GC-MS	50–1000 mg L^−1^	7.00 mg L^−1^	[55]
-	GC-MS	5–100 ng/mL	1.25 ng/mL	[56]
-	UV visible spectrometry	1.00–20.0 mg L^−1^	0.400 mg L^−1^	[57]
-	HPLC	-	LOD: 0.01 ng/μL LQD: 0.03 ng/μL	[58]
-	HPLC	18 to 200 ng/mL	18 ng/ml	[53]

GCE: glassy carbon electrode; EIS: electrochemical impedance spectroscopy; DPV: differential pulse voltammetry; PVC: polyvinylchloride; CPE: carbon paste electrode; SWV: square wave voltammetry; LLOQ: lower limit of quantification; GC–MS: gas chromatography–mass spectrometry; HPLC: high-pressure liquid chromatography; LOD: limit of detection; LQD: limit of quantification.

### 3.4. Reproducibility, Stability, and Selectivity

One of the important features in the design and manufacture of electrochemical sensors is reproducibility. To check reproducibility, the desired electrode was made and examined 10 times in optimized conditions to assess possible fluctuations in the final output of the nanobiosensor. The reproducibility (RSD%) of the optimized nanobiosensor was 3.27% based on those 10 independent tests, suggesting good reproducibility of the fabrication method.

In order to assess stability, biosensors (n = 10) were stored at 4 °C in a refrigerator for two weeks. Their performance was then assessed and compared with that of a freshly prepared nanobiosensor. Accordingly, the signal strength of the electrochemical biosensor remained at 92.9% of its original value, indicating long-term stability.

**Figure 8:**
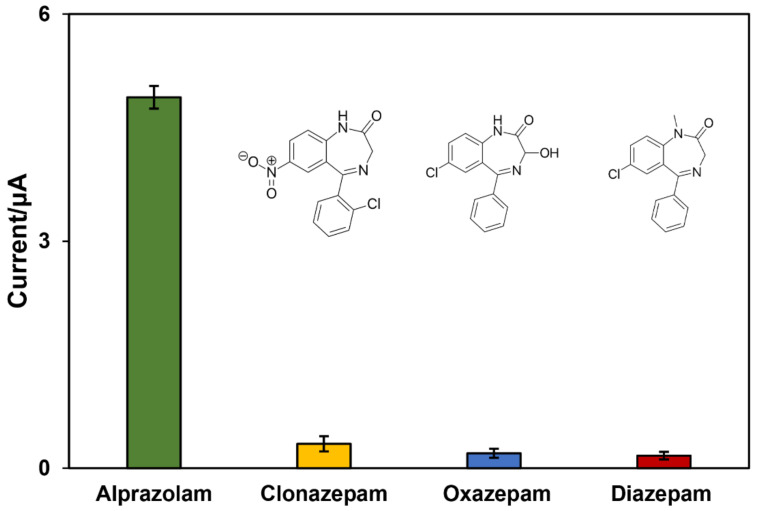
Selectivity of the fabricated nanobiosensor in alprazolam detection in comparison to similar drugs: clonazepam, oxazepam, and diazepam (n = 5).

As for selectivity and interference studies, the output signals of alprazolam (1.0 mg/L) were compared to those of the same concentration of three other drugs with similar chemical structures (clonazepam, diazepam, and oxazepam). In addition, to simulate real samples, three compounds that can be normally found in the blood (glucose, ascorbic acid (Vitamin C), and citric acid) were added to the medium. Those interfering molecules were added at a much higher concentration than the concentration of the target molecule (glucose 40-fold, ascorbic acid 60-fold, and citric acid 50-fold higher). The threshold of interference was considered to be five changes in output signal of alprazolam. Figure 8 shows that the peak current of the alprazolam was significantly higher than that of the other three drugs, representing high selectivity of the sensor even in the presence of interfering molecules such as glucose, ascorbic acid (Vitamin C), and citric acid. As mentioned before, each drug was measured with our nanobiosensor five times, and the low error bars demonstrated that the results are repeatable.

### 3.5. Real Sample

The fabricated biosensor was finally tested in a clinical environment using real samples. Different amounts of alprazolam powder were added to diluted serum samples taken from volunteers who were not taking alprazolam (healthy individuals). Table 2 shows the comparison between different amounts of alprazolam added (spiked) to the real samples and calculated recovery percentage values for each concentration. A recovery rate from 94% to 105.5% (close to 100%), with a low corresponding RSD of 0.8% to 1.7%, was reported for the nanobiosensor based on the assessment of three replications. According to those results, the nanobiosensor was able to detect alprazolam content of real samples with high presented recovery and low SD, illustrating high efficiency of the nanobiosensor in direct spotting of alprazolam in serum samples.

## 4. Conclusions

In this study, we developed an electrochemical nanobiosensor based on a combination of two different nanomaterials (Fe-Ni@rGO and AuNUs) and specific electrochemical pretreatment. The nanobiosensor showed great potential for measuring a wide range of alprazolam concentrations in both synthetic (buffer) and real sample environments (blood serum).

In addition to increasing the surface area of the electrode, the selected nanomaterials also improved its conductivity, which helped facilitate the transfer of electrons to the electrode surface. Furthermore, electrochemical pretreatment of the surface with nanomaterials increased –OH and -COOH groups on Fe-Ni@rGO and the nanobiosensor as a whole. As a result, alprazolam molecules were more likely to be absorbed on the electrode surface, which helped improve the sensor’s performance. The sensor had two linear ranges (4 to 500 µg L^−1^ and 1 to 50 mg L^−1^) and exhibited high sensitivity, low limit of detection (1 µg L^−1^), and good repeatability. The sensor showed promising results in spiked real samples, with low interference with similar drugs (benzodiazepines, clonazepam, oxazepam). The simple and low-cost fabrication of the nanobiosensor, along with its superior sensitivity and selectivity, could make it a proper choice for detection of alprazolam in future clinical applications. More studies in this regard, especially on upscaling-compatible fabrication methods for this type of electrochemical electrode, are required.

## Figures and Tables

**Table 2:** Difference between spiked alprazolam solution in serum and the actual amount with recovery and respective RSD percentages.

Sample	Added	Found	Recovery%	RSD%
1	10 μg/L	9.4 (±0.4) μg/L	94	1.7
2	100 μg/L	102 (±0.8) μg/L	102	0.9
3	1 mg/L	0.98 (±0.03) mg/L	98	0.8
4	20 mg/L	21.1 (±0.2) mg/L	105.5	1.2

## Data Availability

Not applicable.

## Supplementary Materials

The following supporting information can be downloaded at: https://www.mdpi.com/article/10.3390/bios12110945/s1, Figure S1: Full Factorial Design for four nanomaterials Fe-Ni@rGO (F_1_), CdSQD (F_2_), GQD-Ag (F_3_) and AuNUs (F_4_); (A) Pareto chart, (B) Normal probability Plot, (C) Versus Fits, (D) Histogram, (E) Versus Order.
