# Supplementary material for: Alprazolam Detection Using an Electrochemical Nanobiosensor Based on AuNUs/Fe-Ni@rGO Nanocomposite
Source: Biosensors (Basel). 2022 Oct 31;12(11):945. doi: 10.3390/bios12110945 (PMC9687846; doi:10.3390/bios12110945)
Supplement: Supplementary file 1 [file biosensors-12-00945-s001.zip › biosensors-1818770-supplementary.pdf]

Supplementary Information

Alprazolam detection using an electrochemical nanobiosensor based on AuNUs / Fe-Ni@rGO nanocomposite

S1. DOE results:

The full factorial design (FFD) method was thus used to select the best combination of nanomaterials. In this technique, experimental points are created using all possible combinations of levels of factors in every replication or trial. The Pareto charts were used to assess the impact of different materials on the final signal in the electrode manufacturing process and the optimization study. These charts plot the factors with a standardized effect beyond the 95% confidence level (beyond the line at 2.20). The effect of four tested nanomaterials, namely Fe-Ni@rGO (F_1), CdSQD (F_2), GQD-Ag (F_3), and AuNUs (F_4) on the final signal are shown in **Figure S1**. **Figure S1A** plots the absolute values of the influential factors along with a reference line. The effect of the factors passing beyond the reference line is considered potentially significant. Among the four tested nanomaterials, Fe-Ni@rGO and AuNUs crossed the reference line, confirming to play a critical role in the final signal. In the normal probability diagram (**Figure S1B)**, the data are plotted against a theoretical normal distribution to form an approximate straight line. In this figure, the sorted data plotted against the remainder showed a normal distribution, confirmed by the absence of any departures from the straight line. To verify the accuracy of the results and to detect possible unequal error variances or outliers, a residual versus fit diagram was plotted (**Figure S1C**). In this regard, the residual plot shows the difference between the observed response and the fitted response values. The irregular and random scattering of the residuals on both sides of the X-axis confirms good fitting. As shown in Figure 2C, there is no trend for the residuals as expected. The bell-shaped histogram in **Figure S1D** shows the normal distribution of the residuals. The residuals versus order plot (**Figure S1E),** helps assess possible correlations between error terms close to each other in the sequence by examining data in a time (or space) sequence. No trend, however, was seen in the independent residuals.

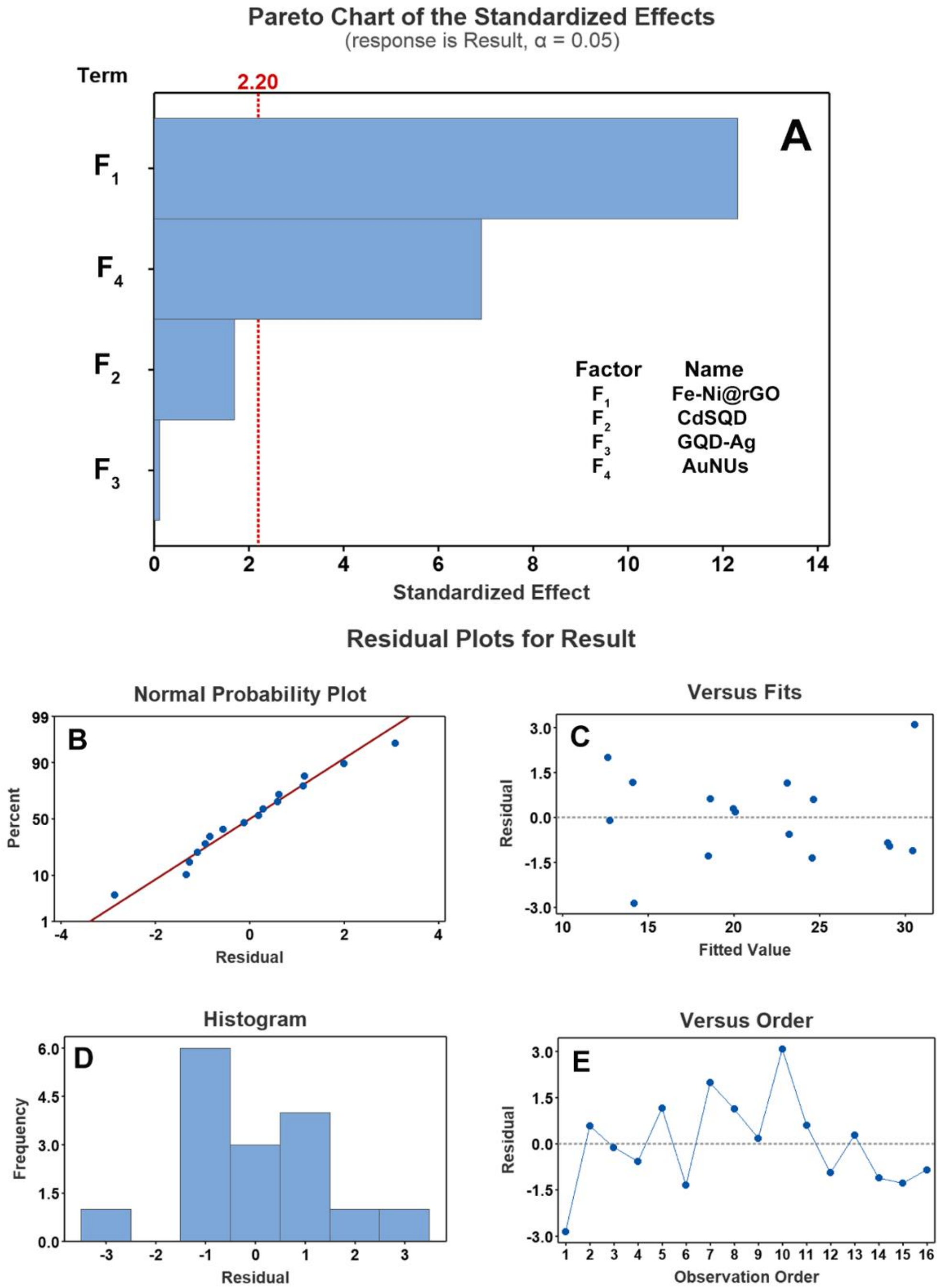

Figure S1. Full Factorial Design for four nanomaterials Fe-Ni@rGO (F_1), CdSQD (F_2), GQD-Ag (F_3) and AuNUs (F_4); (A) Pareto chart, (B) Normal probability Plot, (C) Versus Fits, (D) Histogram, (E) Versus Order.
